# Relação entre a Complacência Pulmonar Estática e a Falha de Extubação em Pacientes Pós-Operatório de Cirurgia Cardíaca

**DOI:** 10.36660/abc.20230350

**Published:** 2024-02-16

**Authors:** Thais Bento Rudge Ramos, Luciana Castilho Figueiredo, Luiz Claudio Martins, Antonio Luis Eiras Falcão, Lígia dos Santos Roceto Ratti, Orlando Petrucci, Desanka Dragosavac

**Affiliations:** 1 Universidade Estadual de Campinas Campinas SP Brasil Universidade Estadual de Campinas, Campinas, SP – Brasil

**Keywords:** Complacência Pulmonar, Cuidados Pós-Operatórios, Cirurgia Cardíaca

## Abstract

**Fundamento:**

Pouco explorada na decisão de extubação no pós-operatório de cirurgia cardíaca, a complacência pulmonar estática seriamente afetada no procedimento cirúrgico pode levar à insuficiência respiratória e à falha na extubação.

**Objetivo:**

Avaliar a complacência pulmonar estática no pós-operatório de cirurgia cardíaca e relacionar sua possível redução aos casos de falha na extubação dos pacientes submetidos ao método *fast-track* de extubação.

**Métodos:**

Foram incluídos pacientes que realizaram cirurgia cardíaca com uso de circulação extracorpórea (CEC) em um hospital universitário estadual admitidos na UTI sob sedação e bloqueio residual. Tiveram sua complacência pulmonar estática avaliada no ventilador mecânico por meio do software que utiliza o *least squares fitting* (LSF) para a medição. No período de 48 horas após a extubação os pacientes foram observados respeito à necessidade de reintubação por insuficiência respiratória. O nível de significância adotado para os testes estatísticos foi de 5%, ou seja, p<0,05.

**Resultados:**

Obtiveram sucesso na extubação 77 pacientes (75,49%) e falharam 25 (24,51%). Os pacientes que falharam na extubação tiveram a complacência pulmonar estática mais baixa quando comparados aos que tiveram sucesso (p<0,001). Identificamos o ponto de corte para complacência por meio da análise da curva *Receiver Operating Characteristic Curve* (ROC) sendo o ponto de corte o valor da complacência <41ml/cmH2O associado com maior probabilidade de falha na extubação (p<0,001). Na análise de regressão múltipla, verificou-se a influência da complacência pulmonar (dividida pelo ponto de corte da curva ROC) com risco de falha 9,1 vezes maior para pacientes com complacência <41ml/cmH2O (p< 0,003).

**Conclusões:**

A complacência pulmonar estática <41ml/cmH2O é um fator que compromete o sucesso da extubação no pós-operatório de cirurgia cardíaca.

## Introdução

A falha na extubação está associada a maior morbimortalidade, maior tempo de ventilação mecânica e retardo da recuperação do paciente, prolongando sua permanência hospitalar. De 4% a 20% dos pacientes extubados falham na extubação, ou seja, são reintubados nas primeiras 42 a 72 horas pós-extubação.^1^ Segundo estudos, a falha na extubação em pacientes submetidos a cirurgia cardíaca pode ser prevista por diversas variáveis como a história médica pregressa dos pacientes, dados laboratoriais, índice de respiração rápida e superficial (RSBI), variáveis cirúrgicas como complexidade e duração da cirurgia e tempo de circulação extracorpórea (CEC).^2,3^ No entanto, outros preditores potenciais não foram adequadamente considerados, sendo generalistas e pouco consensuais os protocolos utilizados para identificar se o paciente estava apto para assumir e manter a ventilação de maneira adequada.^4^

**Figure:**
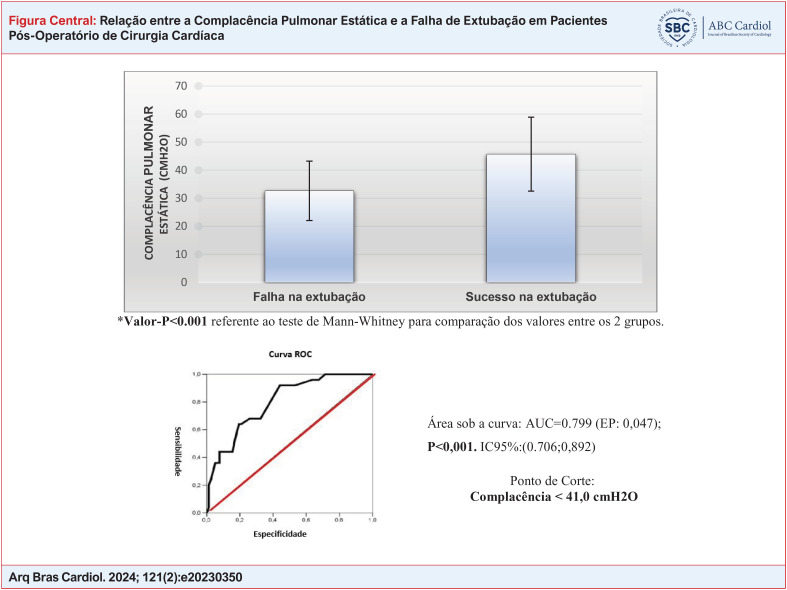
Comparação entre a complacência pulmonar estática e a falha de extubação em pacientes em pós-operatório de cirurgia cardíaca. Ponto de corte para a complacência pulmonar como preditor de falha de extubação.

A partir do início dos anos 1990, os pacientes pós-cirurgia cardíaca passaram a ser extubados tão logo se estabilizasse a hemodinâmica, o nível de consciência e a capacidade ventilatória espontânea. O método conhecido como *“fast track”* visa reduzir as complicações pulmonares ligadas à ventilação mecânica, acelerar a recuperação, diminuir o tempo de permanência na UTI e os custos hospitalares.^5^ Não há na literatura um consenso em relação ao tempo para a extubação, o período varia de duas horas até doze horas após o fim da cirurgia, outros estudos defendem a extubação ainda no centro cirúrgico, o que ficou conhecido como *“ ultra-fast-track”*.^6^ O sucesso da extubação tem lugar pela manutenção da ventilação espontânea pelo período de 48 horas após a extubação.^7^

A cirurgia cardíaca, sobretudo com uso da CEC, compromete o sistema pulmonar podendo causar atelectasia, infecções pulmonares e diminuição da força muscular respiratória, isso consequentemente reduz a complacência pulmonar estática, a capacidade ventilatória e compromete a adequada troca gasosa.^8^

Na literatura encontramos estudos pouco consistentes que norteiem a avaliação da mecânica pulmonar no pós-operatório de cirurgia cardíaca e apontem sua repercussão no resultado da extubação.^9^ O objetivo deste estudo é avaliar o comportamento da mecânica pulmonar no pós-operatório de cirurgia cardíaca por meio da complacência pulmonar estática e relacionar sua possível redução aos casos de falha na extubação dos pacientes submetidos ao método *fast-track*.

## Métodos

### Implicações éticas do estudo

Essa pesquisa está devidamente aprovada pelo Comitê de Ética e Pesquisa (CEP) (Parecer número: 1.867.312) e em conformidade com a resolução 466/12 do Conselho Nacional de Saúde (CNS). Ao se enquadrar nos fatores de inclusão da pesquisa, o paciente foi informado sobre sua realização e assinou o termo de Consentimento Livre e Esclarecido (TCLE) antes da cirurgia. Todas as decisões referentes ao tratamento clínico dos pacientes foram realizadas pelos médicos responsáveis, sem interferência dos pesquisadores.

### Pacientes

**Figura 1:**
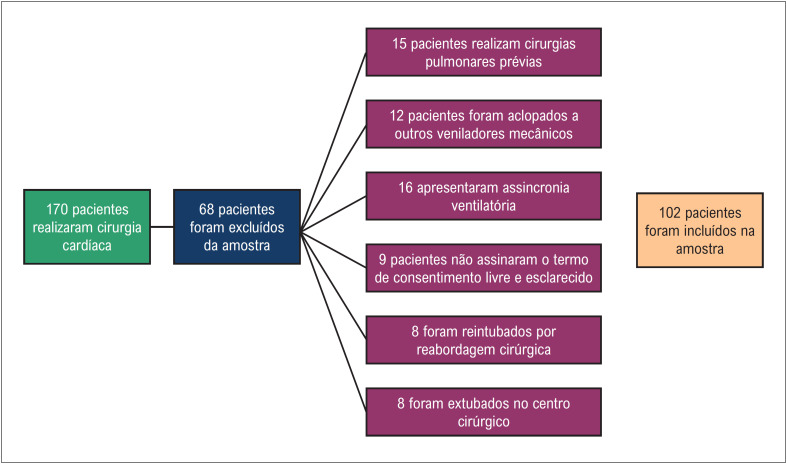
Esquema da seleção da amostra. TCLE: Termo de consentimento livre e esclarecido.

No período de agosto de 2017 a agosto de 2019, 170 pacientes foram submetidos à cirurgia cardíaca em um hospital universitário estadual, destes, 68 pacientes não preencheram os critérios de inclusão (Figura 1). Foram selecionados para o estudo 102 pacientes com idade maior ou igual a 18 anos, de ambos os sexos que realizaram a cirurgia cardíaca eletiva com utilização de CEC, encaminhados para a UTI, sob sedação e bloqueio neuromuscular residual, intubados e acoplados ao ventilador mecânico *Hamilton Raphael Silver*^®^, com parâmetros ventilatórios ajustados previamente, seguindo as diretrizes da instituição: Modalidade assisto-controlada a volume: Volume corrente: 500; Frequência ventilatória: 12; Tempo inspiratório: 1:2; Fio2 50%, PEEP de 5, sem incursões respiratórias espontâneas ou sinais de desconforto respiratório, com dados completos em seu prontuário clínico, pacientes estáveis hemodinamicamente (pressão arterial média entre 70 e 110 mmHg; frequência cardíaca < 90 bpm, índice cardíaco > 1,8 l/min/m²; pressões de enchimento normais, afebril, com diurese presente), submetidos à extubação no período de até 8 horas após a admissão. Foram excluídos da amostra pacientes que não assinaram o TCLE, não foram acoplados ao ventilador mecânico utilizado na pesquisa, foram submetidos a reintubação devido à reabordagem cirúrgica, apresentavam instabilidade hemodinâmica, foram extubados no centro cirúrgico e aqueles que sofreram cirurgias pulmonares e pleurais previas como: lobectomia total, pneumectomia, pleuroscopia ou toracoscopia.

Fonte: Autor

### Coleta e monitoramento dos dados

A coleta e organização dos dados foram efetuadas por meio de uma ficha elaborada pelos pesquisadores, avaliada e aprovada pelo comitê de ética em pesquisa do Hospital. As variáveis coletadas no prontuário médico envolvem dados demográficos como: Número de matrícula, idade, gênero, peso, altura índice de massa corpórea (IMC), tabagismo, *Acute Physiology and Chronic Health disease Classification System II (APACHE II), European System for Cardiac Operative Risk Evaluation(EUROSCORE)* e o *Sequential Organ Failure Assessment (SOFA)* de admissão, dados referentes à cirurgia como: tipo de cirurgia realizada, tempo de CEC, necessidade de hemoderivados, PaO2, SpO2 e relação PaO2/Fio2. Em referência à extubação, foi coletado o tempo de ventilação mecânica após a cirurgia e em caso de reintubação dados referentes à data, horário, quanto tempo após a extubação ocorreu a reintubação, causa da reintubação, tempo de permanência do paciente na ventilação mecânica e informação do desfecho referente à alta, ou óbito. O valor da complacência pulmonar estática foi coletado diretamente no ventilador acoplado ao paciente no momento de sua admissão na UTI com ele já monitorado e estável hemodinamicamente, em decúbito dorsal e cabeceira elevada a 30º. O ventilador mecânico *Hamilton Raphae Silverl*^®^ realizou a medição da complacência estática ventilatória em toda incursão respiratória em todos os modos mandatórios, sem a necessidade de interrupção na ventilação, fluxo inspiratório especial ou padrões e manobras de oclusão, isso devido a seu software que utiliza o método estatístico chamado *least squares fitting* (LSF) para a medição.^10^ Previamente à extubação foi avaliado o índice de oxigenação pulmonar por meio da gasometria arterial. No período de 48 horas após a extubação, os pacientes foram observados respeito as suas condições ventilatórias e necessidade de reintubação por insuficiência respiratória.

Coletadas as variáveis necessárias para análise, descrevemos a casuística comparando as variáveis sociodemográficas, clínicas, cirúrgicas e pulmonares em dois grupos: pacientes que falharam *versus* os que obtiveram sucesso na extubação. Avaliamos um ponto de corte para a complacência pulmonar estática que melhor discriminasse os pacientes que falharam ou não na extubação, e por fim comparamos as variáveis entre os pacientes de acordo com o ponto de corte obtido para a complacência pulmonar.

### Análise estatística

Para descrever o perfil da amostra segundo as variáveis em estudo, foram feitas tabelas de frequência das variáveis categóricas, com valores de frequência absoluta (n) e percentual (%), e estatísticas descritivas das variáveis contínuas, em mediana e intervalo interquartil. Para comparação das variáveis categóricas entre os 2 grupos (com e sem falha) foi utilizado o teste Qui-Quadrado de Pearson, ou o Teste exato de Fisher, na presença de valores esperados menores que 5%. Para comparação das variáveis numéricas entre os 2 grupos foi utilizado o Teste de Mann-Whitney, devido à ausência de distribuição normal da maioria das variáveis. O teste de Kolmogorov-Smirnov foi usado para verificar a normalidade dos dados. Para avaliar um ponto de corte para a complacência pulmonar que melhor discriminasse entre os pacientes com e sem falha de extubação foi usada a análise da curva ROC. Para estudar os fatores relacionados com a falha de extubação foi usada a Análise de regressão de Cox, univariada e múltipla, com critério *Stepwise* de seleção de variáveis. O nível de significância adotado para os testes estatísticos foi de 5%, ou seja, p<0.05.

## Resultados

### Visão geral da população estudada

Foram incluídos na amostra 102 indivíduos. Suas características demográficas clínicas e cirúrgicas são apresentadas na Tabela 1. A população é formada por maioria do sexo masculino com média de idade de 59 anos, não tabagistas e apresentando sobrepeso. Os procedimentos mais realizados foram a revascularização do miocárdio, seguido das trocas valvares. A necessidade de hemoderivados (plasma fresco congelado e concentrado de hemácias) ocorreu na maioria dos casos. A probabilidade de óbito do paciente na sua primeira hora na UTI verificado pelo score SAPS foi mediana, assim como o índice de gravidade desse paciente nas primeiras 24 horas de internação verificado pelo score APACHE III que indicou uma probabilidade de óbito de 15%. No Score SOFA realizado no momento da admissão do paciente na UTI, todos apresentaram algum grau de disfunção orgânica. Em relação às variáveis ventilatórias, o índice de oxigenação pulmonar verificado por meio do cálculo da Pao2/Fio2 foi considerado moderado de acordo com a classificação das Recomendações Brasileiras de Ventilação Mecânica de 2013.^7^ A média da complacência pulmonar estática verificada na admissão do paciente na UTI foi de 42,55 cmH2O.

**Tabela 1:** Características demográficas, clínicas, cirúrgicas e ventilatórias da população do estudo (n=102)

Variável	Resultado
Sexo masculino % (n)	56,86 (58)
Sexo feminino % (n)	43,14 (44)
Idade (anos)	61,50 (52,00-69,00)
Média do Indice de massa corpórea (Kg/m²)	25,30 (22,60-28,40)
Tabagistas % (n)	47,06 (48)
Não Tabagistas % (n)	52,94 (54)
**Dados cirúrgicos**	
Revascularização do miocárdio % (n)	48,04 (49)
Troca de válvula aórtica % (n)	16,67 (17)
Troca de válvula mitral % (n)	13,73 (14)
Transplante cardíaco % (n)	6,86 (7)
Prótese aórtica % (n)	8,82 (9)
Fechamento de comunicação intra valvar	2,94 (3)
Outras cirurgias % (n)	3,00 (3)
Tempo de circulação extracorpórea (min)	88,50 (104,00-75,00)*
Necessidade de hemoderivados % (n)	52,94% (54)
**Scores**	
APACHE	13,00 (16,00-10,00)
SAPS*	38,00 (32,00-43,00)
SOFA* de admissão	5,00 (4,00-7,00)
**Dados Ventilatórios**	
Relação ventilação perfusão ( Pao2/ Fio2)	169,00 (145,00-243,00)
Complacência pulmonar estática ( cmH2O)	40,00 (35,00-49,00)

APACHE II: Acute Physiology and Chronic Health disease Classification System; SOFA: Sequential Organ Failure System; SAPS: Simplified Acute Physiology Score. Teste estatístico Kolmogorov-Smirnov foi usado para verificar a normalidade dos dados; valor de referência p<0.05. Descrição: mediana e intervalo interquartil.

**Figura 2:**
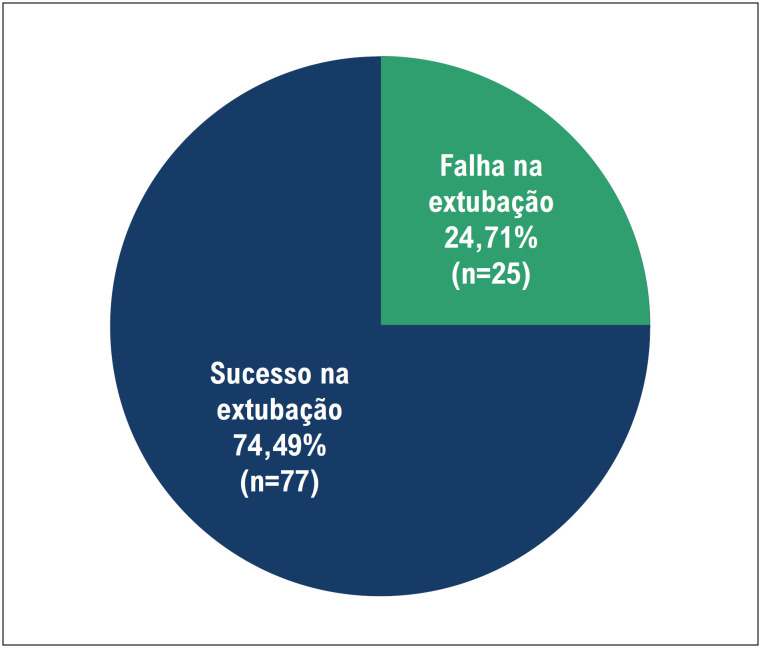
Gráfico referente ao desfecho nas extubações, entre os dois grupos: sucesso e falha na extubação no período de 48 horas.

Observamos na Figura 2 a proporção entre os grupos de sucesso e de falha na extubação.

Fonte: Autor

### Análise comparativa entre os grupos: Falha na extubação versus Sucesso na extubação

A Tabela 2 fornece a comparação das variáveis demográficas, clínicas, e cirúrgicas entre os dois grupos estudados. Pelos resultados, se verificou diferença significativa entre os com e sem falha de extubação no sexo feminino, que apresentou mais falha do que o masculino. Os pacientes reintubados apresentaram de média a alta probabilidade de óbito na aplicação dos escores APACHE e SAPS e com elevada disfunção orgânica predita pelo escore SOFA.

**Tabela 2:** Comparação categórica e numérica das características demográficas, cirúrgicas e clínicas entre sucesso versus falha na extubação

Variáveis		Falha da extubação (n=25)	Sucesso da extubação (n=77)	Valor-p*
		% (n)	% (n)	
Sexo Masculino		32,00 (8)	35,06 (27)	
Sexo Feminino		68,00 (17)	64,94 (50)	
Teste Qui-quadrado: X2=8,35; GL=1; p=0,004				
Tabagistas		60,00 (15)	42,86 (33)	
Não tabagistas		40,00 (10)	57,14 (44)	
Teste Qui-quadrado: X2=2,23; GL=1; p=0,136				
Necessidade de hemoderivados		64,00 (16)	49,35 (38)	
Teste Qui-quadrado: X2=2,23; GL=1; p=0,136				
Cirurgia realizada				
Revascularização do miocárdio		36,00** (**9)	51,95** (**40)	
Troca válvula aórtica		20,00 (5)	15,58 (12)	
Troca válvula mitral		12,00 (3)	14,29 (11)	
Transplante cardíaco		8,00 (2)	6,49 (5)	
Prótese de aorta		16,00 (4)	6,49 (5)	
Teste exato de Fisher: p=0,463				
Idade (anos)		62,00 (53,00-69,00)	61,00 ( (52,00-68,00)	0,864
Indice de massa corpórea (Kg/m²)		25,50 (23,80-26,40)	25,00 (22,40-28,70)	0,741
	APACHE II	15,00 (12,00-18,00)	12,00 (9,00-14,00)	0,004
	SAPS III	41,00 (38,00-47,00)	37,00 (31,00-42,00)	0,027
SOFA admissão		6,00 (5,00-8,00)	5,00 (3,00-6,00)	0,011
Tempo de CEC (min)		91,00 (70,00-104,0)	88,00 (78,00-103,0)	0,861

Valor-p referente ao teste de Mann-Whitney para comparação dos valores entre os 2 grupos. APACHE II: Acute Physiology and Chronic Health disease Classification System; SOFA: Sequential Organ Failure System; SAPS: Simplified Acute Physiology Score.

A Tabela 3 e a Figura Central apresentam a comparação dos dois grupos quanto ao valor da complacência pulmonar estática, sendo este significantemente reduzido nos pacientes que falharam na extubação. Quanto à capacidade pulmonar de oxigenação, os dois grupos apresentam valores que indicam oxigenação moderada.

**Tabela 3:** Variáveis pulmonares entre falha e sucesso de extubação

Variáveis	Falha na extubação	Sucesso na extubação	Valor-p*
Complacência pulmonar estática	35,00 (30,00-40,00)	43,00 ( 38,00-43,00)	<0,001
Relação Pao2/Fio2	156,0 (245,0-196,0)	183,0 (148,0-247,0)	0,161

* Valor-p referente ao teste de Mann-Whitney para comparação dos valores entre os 2 grupos. Relação PaO2/ Fio2: Pressão parcial de oxigênio pela fração inspirada de oxigênio.

### Análise da curva ROC para a complacência pulmonar estática *versus* falha na extubação

**Figura 3:**
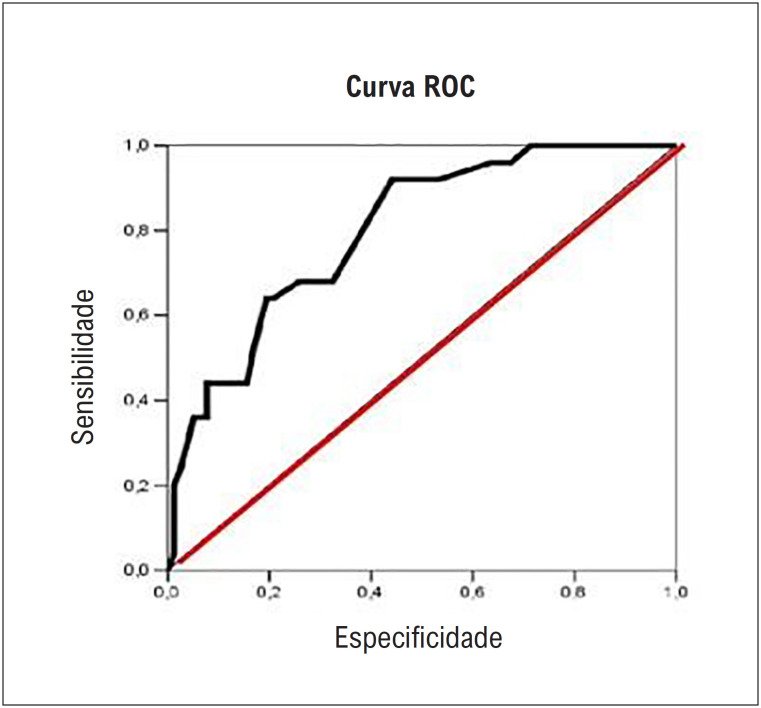
Área sob a curva: AUC=0,799 (EP: 0,047); p<0,001. IC95%: (0,706; 0,892) Ponto de Corte: Complacência < 41,0cmH2O. Fonte: Próprio autor

A Tabela 4 fornece o resultado da análise da curva ROC (*Receiver Operating Characteristic curve*) (Figura 3) para avaliar um ponto de corte para a complacência pulmonar como preditor de falha de extubação. Pelos resultados, verificou-se que a complacência pulmonar obteve área sob a curva significativa, sendo o ponto de corte o valor da complacência <41,0 cmH2O associado com maior probabilidade de falha de extubação.

**Tabela 4:** Resultado da análise da curva ROC para complacência pulmonar versus falha de extubação

Complacência pulmonar estática <41ml/cmH2O			
Variáveis	Falha na extubação	Sucesso na extubação	Total
% (n)	22,55% (23)	33,33% (34)	55,88% (57)
Ponto de corte (Linha)	40,35	59,65	
Probabilidade de falha (Coluna)	92,00	44,16	
**Complacência pulmonar estática ≥41ml/cmH2O**			
% (n)	1,96% (2)	42,16% (43)	44,12 (45)
Ponto de corte (Linha)	4,44	95,56	
Probabilidade de falha (Coluna)	8,00	55,84	

Sensibilidade (IC95%): 92,00% (72,50; 98,60) Especificidade (IC95%): 55,84% (44,10; 67,00) Valor preditivo positivo (IC95%): 40,35% (27,84; 54,16) Valor preditivo negativo (IC95%): 95,56% (83,64; 99,23) Acurácia (IC95%): 64,71% (54,55; 73,74) .Teste Qui-Quadrado: X2=17,52; GL=1; p<0,001.

* ROC= Receiver Operating Characteristic Curve

### Análise de Regressão de Cox para a falha na extubação

As Tabelas 5 e 6, a seguir, apresentam os resultados das análises de Regressão de Cox para estudar os fatores relacionados com a falha de extubação. Inicialmente, foi realizada a análise univariada, e depois a análise múltipla com critério *Stepwise* de seleção.

**Tabela 5:** Resultados da análise de regressão de Cox univariada para falha de extubação (n=102)

Variável	Categorias	Valor-p	H.R.*	IC 95% H.R.*
Idade	Variável contínua	0,731	1,006	0,974 – 1,039
Gênero	Masculino (ref.)	---	1.00	---
	Feminino	0,016	2,80	1,21 – 6,49
IMC	Variável contínua	0,950	1,002	0,936 – 1,073
Relação PaO2/FiO2	Variável contínua	0,235	0,996	0,990 – 1,002
Tempo CEC	Variável contínua	0,916	0,999	0,981 – 1,017
Complacência pulmonar	Variável contínua	<0,001	0,953	0,929 – 0,977
Complacência pulmonar (ponto de corte curva ROC)	≥41 (ref.)	---	1,00	---
	<41	0,003	9,08	2,14 – 38,50

* HR (Hazard Ratio): Razão de risco para falha; (n=77 sem falha e n=25 com falha). IC95% HR: Intervalo de 95% de confiança para a razão de risco. Ref.: nível de referência. IMC: índice de massa corpórea; Relação PaO2/ Fio2: pressão parcial de oxigênio pela fração inspirada de oxigêni; CEC: circulação extracorpórea; ROC: Receiver Operating Characteristic Curve.

**Tabela 6:** Resultados da análise de regressão de Cox múltipla para falha de extubação (n=102)

Variável	Categorias	Valor-p	H.R.*	IC 95% H.R.*
1. Complacência pulmonar(ponto de corte curva ROC)	≥41 (ref.)	---	1,00	---
	<41	0,003	9,08	2,14 – 38,50

* HR (Hazard Ratio): Razão de risco para falha; (n=77 sem falha e n=25 com falha). IC 95% HR: Intervalo de 95% de confiança para a razão de risco. Critério Stepwise de seleção de variáveis. Ref.: nível de referência.

Pelos resultados da análise múltipla, verificou-se a influência do seguinte fator na falha de extubação: a complacência pulmonar (dividida pelo ponto de corte da curva ROC) apresenta risco de falha 9,1 vezes maior para os pacientes com complacência < 41 cmH2O.

## Discussão

A falha na extubação está entre as complicações frequentes na unidade de terapia intensiva. Segundo o estudo de Ruan et al., 10% das extubações falham e há a necessidade de retorno à ventilação mecânica nas primeiras 48 horas.^11^ A falha na extubação eleva substancialmente a probabilidade de óbito, além de prorrogar o período de internação na UTI. Scott et al. em seu estudo, concluem que pacientes que falham na extubação têm sete vezes mais chance de evoluir a óbito e são 31 vezes mais propensos a ter uma internação prolongada quando comparados a pacientes que tiveram sucesso na extubação.^12^ Em nosso estudo, 77 pacientes (75,49%) tiveram sucesso na extubação, enquanto falharam 25 (24,51%), estes índices de sucesso e falha se assemelham a outros estudos como o de Danaga et al. que avaliaram o índice de falha de extubação em uma amostra de 73 pacientes, onde 58 (80,0%) permaneceram extubados e 15 (20,0%) falharam e necessitaram retornar à ventilação mecânica.^13^ Souza e Lugon avaliaram 109 pacientes, 65 pacientes (59,6%) foram extubados com sucesso porem 36 (33%) foram a óbito (8 pacientes já haviam sido extubados), a taxa de reintubação nesse estudo foi de 10,7%.^14^ Ainda que tenhamos mais sucesso do que falhas nas extubações, as consequências da reintubação são graves visto o alto índice de morbimortalidade.

A amostra do estudo foi composta predominantemente por homens, característica que acompanha a tendencia de outros estudos como o de Dordetto et al. que avaliaram as características demográficas de 100 pacientes submetidos a cirurgia cardíaca, deles 56,0% eram homens e 44,0% mulheres.^15^ Isso pode ser justificado pelo fato das doenças cardiovasculares serem mais letais em mulheres. Segundo a Organização Mundial da Saúde (OMS) (2018) as cardiopatias correspondem a um terço das causas de morte em mulheres no mundo.^16^ Isto é devido ao fato de que os sintomas indicativos de doenças cardiovasculares são mais genéricos nas mulheres dificultando o diagnóstico para iniciar o tratamento e impedindo que elas cheguem à cirurgia cardiovascular.^17^ Apesar da maioria masculina no procedimento, o estudo apontou que as mulheres falharam mais na extubação do que os homens, esse fato pode ser justificado pelo estudo de Pereira et al. que apontaram um padrão respiratório na mulher predominantemente torácico, isso é por ter as costelas superiores mais móveis permitindo assim uma maior expansão apesar de ter a capacidade torácica menor devido ao esterno mais curto e a abertura torácica superior mais oblíqua.^18^ Sendo assim, devido a toracotomia com ou sem a incisão pleural, a intensidade da manipulação cirúrgica e a quantidade de drenos pleurais, as mulheres tem sua expansibilidade torácica mais comprometida, Ambrozin et al. que pesquisaram os aspectos da função pulmonar em pacientes pós-cirurgia cardíaca, constataram a complacência pulmonar estática reduzida, na maioria em mulheres,^19^ resultando em uma pior troca gasosa, o que as deixa mais susceptíveis à insuficiência respiratória aguda e a falhar na extubação.

A idade média dos pacientes de 58,7 anos não impactou no resultado da extubação. Este fato pode ser justificado pelo estudo de Rocha et al. que concluíram que pacientes com idade acima dos 70 anos são mais propensos a desenvolver complicações respiratórias no pós-operatório de cirurgia cardíaca devido às alterações fisiológicas que ocorrem no processo de envelhecimento, às alterações no tecido conjuntivo que aumentam a rigidez da caixa torácica e reduzem o componente elástico dos pulmões, influenciando a mecânica respiratória além da diminuição da força muscular respiratória, somado a isso, o comprometimento torácico causado pelo procedimento leva essa população a serem mais susceptíveis a complicações.^20,21^

O tipo de cirurgia mais realizada foi a revascularização do miocárdio (48,04%), seguida de trocas valvares (30,43%). Segundo o Data SUS (2018) foram realizadas no Brasil cerca de 23 mil cirurgias cardíacas, compreendendo plastias e trocas valvares e a revascularização miocárdica.^22^ Não encontramos influência do tipo de cirurgia na posterior falha de extubação. No estudo de Assis et. al. que avaliaram a influência do tipo de cirurgia nas complicações pós-operatórias de 57 pacientes, ocorreram apenas duas falhas na extubação, sendo uma em um paciente submetido a revascularização do miocárdio, e a outra em uma revascularização do miocárdio associada a troca valvar,^4^ o que demonstra que o tipo de cirurgia não é um fator predominante para a falha na extubação.

A obesidade torna mais comum a presença de atelectasias nas regiões basais do pulmão reduzindo sua complacência e a presença de tecido adiposo na região torácica aumenta a resistência aérea.^23^ Essa condição repercute de maneira negativa no resultado da extubação, Parlow et al. observaram que os pacientes que preencheram os critérios para obesidade (índice de massa corpórea (IMC) acima de 30 falharam 42,4% mais na extubação do que os pacientes com sobrepeso.^24^ Isso justifica o resultado encontrado em nosso estudo onde a média dos pacientes apresentaram sobrepeso (IMC de 25,91) o que não impactou de maneira significativa no resultado da extubação.

Apesar de não apresentar significância estatística, o tabagismo está relacionado a um maior número de reintubações neste estudo. Ngaage D et al. justificaram esse resultado alegando que tabagistas desenvolvem complicações pulmonares até duas vezes mais do que os não tabagistas ou ex-tabagistas, como consequência, esses pacientes podem permanecer até seis horas a mais em ventilação mecânica no pós-operatório de cirurgia cardíaca.^25^ Possivelmente a extubação precoce dessa população levou a falha na extubação.

Entre os escores comumente usados nas unidades de terapia intensiva para predizer a gravidade do doente e o risco de óbito temos: APACHE II, SAPS e o SOFA de admissão. Observamos que os piores desfechos preditos pelos escores estão ligados aos pacientes que falharam na extubação, esse resultado corrobora o de Shoji et al. que avaliaram o índice de reintubação em pacientes submetidos à cirurgia cardíaca, e constataram que 7,3% dos 119 pacientes avaliados em seu estudo foram reintubados na sua permanência na UTI, desses, 40,3% evoluíram a óbito.^26^

A relação PaO2/FiO2 é utilizada para determinar a capacidade de oxigenação pulmonar do paciente a ser extubado e é um critério que essa relação esteja >200.^19^ Existe um comprometimento da oxigenação em 20 a 90% dos pacientes submetidos à cirurgia cardíaca com CEC.^15^ Em nosso estudo a relação PaO2/Fio2 não influenciou de maneira significativa o desfecho da extubação, porém apresentou-se menor em pacientes que falharam na extubação (178,4 ± 64,14) quando comparados àqueles que tiveram sucesso (203,0 ± 83,00).

A complacência pulmonar estática é um fator importante para a avaliação da mecânica pulmonar, a sua redução aumenta o trabalho respiratório e pode evoluir para uma insuficiência respiratória. O comprometimento da complacência pulmonar na cirurgia cardíaca é um fato consensual entre os estudos relacionados ao assunto e é atribuído a fatores inflamatórios e mecânicos no pré, intra e pós-operatórios.^27^ Segundo o III Consenso de Ventilação Mecânica os valores de complacência considerados normais são de 60 a 100 ml/cmH2O.^15^ Observamos neste estudo que entre os fatores que influenciaram a falha na extubação está a complacência pulmonar estática criticamente reduzida no momento da admissão. Foi possível identificar como ponto de corte o valor de complacência < 41 ml/cmH2O, como o valor associado com 9,1 vezes maior probabilidade de falha na extubação entre os pacientes. Isso pode ser explicado pelo estudo de Cordeiro et al. que avaliaram o impacto da complacência pulmonar na troca gasosa dos pacientes após a cirurgia cardíaca e concluíram que quanto menor a complacência pulmonar estática do paciente pior é a troca gasosa, gerando a insuficiência respiratória aguda, e aumentando a probabilidade de uma reintubação.^28^

Na tentativa de antever o sucesso ou falha na extubação, inúmeras variáveis a serem avaliadas previamente foram propostas ao longo dos anos, porém a disparidade entre os estudos dificulta uma avaliação eficiente para encontrar parâmetros de avaliação confiáveis para prever o sucesso da extubação. Baptistella et al. realizaram uma revisão sistemática que incluiu 43 artigos, desses apenas dois artigos abordaram a complacência pulmonar como um parâmetro a ser utilizado na decisão de extubação e nenhum dos estudos teve na população estudada pacientes após cirurgia cardíaca.^29^ Como é comprovado neste estudo, o comprometimento da complacência pulmonar estática é um fator presente entre os pacientes que falham na extubação, sendo de grande importância inclui-lo na avaliação do paciente pós-operado de cirurgia cardíaca.

### Limitações do estudo

Este estudo teve algumas limitações. O fato de o estudo ter sido realizado em um único hospital, com cirurgias realizadas por uma mesma equipe médica e cuidados pós-operatórios padronizados, pode não refletir a realidade de outros hospitais, com protocolos e condutas distintas. Nem todos os pacientes operados puderam participar da pesquisa ainda que falhassem na extubação posteriormente, devido ao fato de serem extubados no centro cirúrgico e não ser possível a coleta do valor de complacência no ventilador mecânico, isso limitou o tamanho da amostra.

Os pacientes incluídos na nossa amostra não usavam o Cateter Swan-Ganz, o que impossibilitou a avaliação precisa do perfil hemodinâmico e a identificação do choque cardiogênico por exemplo, como fator indicativo de reintubação, contudo, o que temos é que nenhum paciente apresentou sinais clínicos de instabilidade hemodinâmica pré-extubação nos casos estudados.

## Conclusão

Concluímos que a complacência pulmonar estática quando reduzida é um fator de risco para a falha de extubação em pacientes pós-operados de cirurgia cardíaca, sendo o ponto de corte associado a maior falha, a complacência <41ml/cmH2O, sendo assim, demostramos que essa é uma importante variável a ser avaliada previamente à extubação a fim de evitar uma possível reintubação e todos os riscos dela decorrentes.

## References

[1] Ely EW, Meade MO, Haponik EF, Kollef MH, Cook DJ, Guyatt GH (2001). Mechanical Ventilator Weaning Protocols Driven by Nonphysician Health-Care Professionals: Evidence-Based Clinical Practice Guidelines. Chest.

[2] Sanson G, Sartori M, Dreas L, Ciraolo R, Fabiani A. (2018). Predictors of Extubation Failure After Open-Chest Cardiac Surgery Based on Routinely Collected Data. The Importance of a Shared Interprofessional Clinical Assessment. Eur J Cardiovasc Nurs.

[3] Souza LC, Lugon JR (2015). The Rapid Shallow Breathing Index as a Predictor of Successful Mechanical Ventilation Weaning: Clinical Utility When Calculated from Ventilator Data. J Bras Pneumol.

[4] Assis CR, Fortino CK, Souza Saraiva CA, Frohlich LF, da Silva RE, Omizzollo S. (2020). Perfil Clínico e Sucesso na Extubação de Pacientes Pós Cirurgia Cardíaca. Rev Pesq Fisio.

[5] Nogueira TM, Monteiro DS (2010). Fast Track in Heart Surgery: When and How to Perform. Rev Med Minas Gerais.

[6] Nguyen J, Nacpil N. (2018). Effectiveness of Dexmedetomidine Versus Propofol on Extubation Times, Length of Stay and Mortality Rates in Adult Cardiac Surgery Patients: a Systematic Review and Meta-Analysis. JBI Database System Rev Implement Rep.

[7] Barbas CSV, Ísola AM, Farias AMC, Cavalcanti AB, Gama AMC, Duarte ACM (2014). Recomendações brasileiras de ventilação mecânica 2013. Parte Rev I. Bras Ter Intensiva.

[8] Lima CA, Ritchrmoc MK, Leite WS, Silva DARG, Lima WA, Campos SL (2019). Impact of Fast-Track Management on Adult Cardiac Surgery: Clinical and Hospital Outcomes. Rev Bras Ter Intensiva.

[9] Santos WP, Santos PCAB, Diniz FL, Vieira BCB, Oliveira LLC, Ferreira LA (2020). Avaliação do Novo Protocolo de Extubação em Paciente Submetido a Cirurgia Cardiaca. SciGen.

[10] Salomão JM Neto (2010). Ventilador para Terapia Intensiva RAPHAEL: Manual do Operador.

[11] Ruan SY, Teng NC, Wu HD, Tsai SL, Wang CY, Wu CP (2017). Durability of Weaning Success for Liberation from Invasive Mechanical Ventilation: an Analysis of a Nationwide Database. Am J Respir Crit Care Med.

[12] Epstein SK, Ciubotaru RL, Wong JB (1997). Effect of Failed Extubation on the Outcome of Mechanical Ventilation. Chest.

[13] Danaga AR, Gut AL, Antunes LC, Ferreira AL, Yamaguti FA, Christovan JC (2009). Evaluation of the Diagnostic Performance and Cut-Off Value for the Rapid Shallow Breathing Index in Predicting Extubation Failure. J Bras Pneumol.

[14] Souza LC, Lugon JR (2015). Índice de Respiração Rápida e Superficial Como Previsor de Sucesso de Desmame da Ventilação Mecânica: Utilidade Clínica Quando Mensurado a Partir de Dados do Ventilador. J Bras Pneumol.

[15] Dordetto PR, Pinto GC, Rosa TCSC (2016). Pacientes Submetidos à Cirurgia Cardíaca: Caracterização Sociodemográfica, Perfil Clínico-Epidemiológico e Complicações. Rev. Fac. Ciênc. Méd. Sorocaba.

[16] World Organization (2020). Technical package for cardiovascular disease management in primary health care: Risk-based CVD management [Internet].

[17] Schmidt K, Lima ADS, Schmitt KR, Moraes MA, Schmidt MM (2020). Stress in Women with Acute Myocardial Infarction: a Closer Look. Arq Bras Cardiol.

[18] Parreira VF, Bueno CJ, França DC, Vieira DSR, Pereira DR, Britto RR (2010). Breathing Pattern snd Thoracoabdominal Motion in Healthy Individuals: Influence of Age And Sex. Rev Bras Fisioter.

[19] Ambrozin A, Cataneo AJ (2005). Aspectos da Função Pulmonar Após Revascularização do Miocárdio Relacionados com Risco Pré-Operatório. Braz J Cardiovasc Surg.

[20] Rocha ASC, Pittella FJM, Lorenzo AR, Barzan V, Colafranceschi AS, Brito JOR (2012). A Idade Influencia os Desfechos em Pacientes com Idade Igual ou Superior a 70 Anos Submetidos à Cirurgia de Revascularização Miocárdica Isolada. Braz J Cardiovasc Surg.

[21] Lanza FC, Camargo AA, Archija LR, Selman JP, Malaguti C, Dal Corso S. (2013). Chest Wall Mobility is Related to Respiratory Muscle Strength and Lung Volumes in Healthy Subjects. Respir Care.

[22] Brasil. Ministério da Saúde (2018). Sistema de Informações Hospitalares do SUS (SIH/SUS). Número de internações e óbitos por ano/mês processamento segundo região.

[23] Santos NP, Adriana Kathleen Freitas Rocha (2013). Effects of Different Peep Levels on Immediate Postoperative Period in Obese Patients Submitted to Coronary Artery Bypass Graft. Rev Assobrafair Ciência.

[24] Parlow JL, Ahn R, Milne B. (2006). Obesity is a Risk Factor for Failure of “Fast Track” Extubation Following Coronary Artery Bypass Surgery. Can J Anaesth.

[25] Ngaage DL, Martins E, Orkell E, Griffin S, Cale AR, Cowen ME (2002). The Impact of the Duration of Mechanical Ventilation on the Respiratory Outcome in Smokers Undergoing Cardiac Surgery. Cardiovasc Surg.

[26] Shoji CY, Figuereido LC, Calixtre EM, Rodrigues CDA, Falcão ALE, Martins PP (2017). Reintubation of Patients Submitted to Cardiac Surgery: a Retrospective Analysis. Rev Bras Ter Intensiva.

[27] Carvalho CR, Toufen C, Franca SA (2007). Mechanical Ventilation: Principles, Graphic Analysis and Ventilatory Modalities. J Bras Pneumol.

[28] Cordeiro ALL, Oliveira LFL, Queiroz TC, Santana VLL, Melo TA, Guimarães AR (2018). Association of Respiratory Mechanics with Oxygenation and Duration of Mechanical Ventilation After Cardiac Surgery. Int. Cardiovasc J. Sci.

[29] Baptistella AR, Sarmento FJ, Silva KR, Baptistella SF, Taglietti M, Zuquello RÁ (2018). Predictive Factors of Weaning from Mechanical Ventilation and Extubation Outcome: a Systematic Review. J Crit Care.

