# Supplementary material for: Relação entre a Complacência Pulmonar Estática e a Falha de Extubação em Pacientes Pós-Operatório de Cirurgia Cardíaca
Source: Arq Bras Cardiol. 2024 Feb 16;121(2):e20230350. [Article in Portuguese] doi: 10.36660/abc.20230350 (PMC11081412; doi:10.36660/abc.20230350)
Supplement: Translation

# Translation: Relationship between Static Lung Compliance and Extubation Failure in Postoperative Cardiac Surgery Patients

**Authors:** Thais Bento Rudge Ramos, Luciana Castilho Figueiredo, Luiz Claudio Martins, Antonio Luis Eiras Falcão, Lígia dos Santos Roceto Ratti, Orlando Petrucci, Desanka Dragosavac

**Affiliations:** 1 Campinas SP Brasil Universidade Estadual de Campinas, Campinas, SP – Brasil

**Keywords:** Lung Compliance, Postoperative Care, Thoracic Surgery

## Abstract

**Background:**

Static lung compliance, which is seriously affected during surgery, can lead to respiratory failure and extubation failure, which is little explored in the decision to extubate after cardiac surgery.

**Objective:**

To evaluate static lung compliance in the postoperative period of cardiac surgery and relate its possible reduction to cases of extubation failure in patients submitted to the fast-track method of extubation.

**Methods:**

Patients undergoing cardiac surgery using cardiopulmonary bypass (CPB) at a state university hospital admitted to the ICU under sedation and residual block were included. Their static lung compliance was assessed on the mechanical ventilator using software that uses least squares fitting (LSF) for measurement. Within 48 hours of extubation, the patients were observed for the need for reintubation due to respiratory failure. The level of significance adopted for the statistical tests was 5%, i.e., p<0.05.

**Results:**

77 patients (75.49%) achieved successful extubation and 25 (24.51%) failed extubation. Patients who failed extubation had lower static lung compliance compared to those who succeeded (p<0.001). We identified the cut-off point for compliance through analysis of the Receiver Operating Characteristic Curve (ROC), with the cut-off point being compliance <41ml/cmH2O associated with a higher probability of extubation failure (p<0.001). In the multiple regression analysis, the influence of lung compliance (divided by the ROC curve cut-off point) was found to be 9.1 times greater for patients with compliance <41ml/cmH2O (p< 0.003).

**Conclusions:**

Static lung compliance <41ml/cmH2O is a factor that compromises the success of extubation in the postoperative period of cardiac surgery.

## Introduction

Extubation failure is associated with higher morbidity and mortality, longer mechanical ventilation time, and delayed recovery, prolonging the patient's hospital stay. Between 4% and 20% of extubated patients fail extubation, i.e., they are reintubated in the first 42 to 72 hours after extubation.^1^ According to studies, extubation failure in patients undergoing cardiac surgery can be predicted by several variables, such as the patient's previous medical history, laboratory data, rapid shallow breathing index (RSBI), surgical variables such as the complexity and duration of the surgery and cardiopulmonary bypass (CPB) time.^2,3^ However, other potential predictors have not been adequately considered, and the protocols used to identify whether the patient is able to take up and maintain ventilation adequately are still general and not widely agreed upon.^4^

**Figure:** Comparison between static lung compliance and extubation failure in postoperative cardiac surgery patients. Cut-off point for lung compliance as a predictor of extubation failure

Since the early 1990s, post-cardiac surgery patients have been extubated as soon as their hemodynamics, level of consciousness, and spontaneous ventilatory capacity are stabilized. The method known as *“fast track*” aims to reduce pulmonary complications linked to mechanical ventilation, speed up recovery, and reduce ICU stay and hospital costs.^5^ There is no consensus in the literature regarding the time for extubation; the period varies from two hours to twelve hours after the end of the surgery, while other studies advocate extubation while still in the operating room, which has become known as “*ultra-fast-track*”.^6^ Successful extubation is achieved by maintaining spontaneous ventilation for 48 hours after extubation.^7^

Cardiac surgery, especially with the use of CPB, compromises the pulmonary system and can cause atelectasis, lung infections, and decreased respiratory muscle strength, which consequently reduces static lung compliance and ventilatory capacity and compromises adequate gas exchange.^8^

In the literature, we found few consistent studies that guide the evaluation of pulmonary mechanics in the postoperative period of cardiac surgery and point to the repercussions of its impairment on the outcome of extubation.^9^ The objectives of this study are to evaluate the behavior of pulmonary mechanics in the postoperative period of cardiac surgery through static pulmonary compliance and to relate its possible reduction to cases of extubation failure in patients submitted to the *fast-track* method of extubation.

## Methods

### Ethical implications of the study

This research was duly approved by the Research Ethics Committee (CEP) (opinion number: 1.867.312) and complied with resolution 466/12 of the National Health Council (CNS). Once they met the inclusion criteria for the study, the patients were informed about the study and signed the Informed Consent Form (ICF) before the surgery. All decisions regarding the patients’ clinical treatment were made by the doctors in charge, without interference from the researchers.

### Patients

From August 2017 to August 2019, 170 patients underwent cardiac surgery at a state university hospital, 68 of whom did not meet the inclusion criteria (Figure 1). The study included 102 patients aged 18 years or older, of both sexes, who underwent elective cardiac surgery using CPB, referred to the ICU, under sedation and residual neuromuscular blockade, intubated and attached to the Hamilton Raphael Silver^®^ mechanical ventilator, with ventilation parameters previously adjusted, following the institution's guidelines: Volume-controlled assisted mode: Tidal volume: 500; Ventilatory frequency: 12; Inspiratory time: 1:2; Fio2 50%, PEEP of 5, with no spontaneous respiratory incursions or signs of discomfort, with complete data in their clinical record, hemodynamically stable patients (mean arterial pressure between 70 and 110 mmHg; heart rate < 90bpm, cardiac index > 1.8 l/min/m²; normal filling pressures, afebrile, with diuresis present) who underwent extubation within 8 hours of admission. Patients who did not sign the informed consent form, who were not connected to the mechanical ventilator used in the study, who underwent reintubation due to surgical reapproach, with hemodynamic instability, who were extubated in the operating room, and those who had undergone previous lung and pleural surgery such as total lobectomy, pneumectomy, pleuroscopy or thoracoscopy.

**Figure 1:** Sample selection diagram. Caption: ICF: Informed Consent Form.

### Data collection and monitoring

Data was collected and organized using a form drawn up by the researchers, which was assessed and approved by the hospital's research ethics committee. The variables collected from the medical records include demographic data such as enrollment number, age, gender, weight, height, body mass index (BMI), smoking, Acute Physiology and Chronic Health Disease Classification System II (APACHE II), European System for Cardiac Operative Risk Evaluation (EUROSCORE) and Sequential Organ Failure Assessment (SOFA) on admission, data regarding surgery such as type of surgery performed, CPB time, need for blood products, PaO2, SpO2 and PaO2/Fio2 ratio. With regard to extubation, the time spent on mechanical ventilation after surgery was collected. In the event of reintubation, data was collected on the date, time, how long after extubation the reintubation took place, the cause of the reintubation, the length of time the patient remained on mechanical ventilation, and information on the outcome - discharge or death. The static lung compliance value was collected directly from the ventilator attached to the patient on admission to the ICU, with the patient already monitored and hemodynamically stable, in the supine position, head elevated to 30º. The Hamilton Raphae Silverl® mechanical ventilator measures static ventilatory compliance throughout the respiratory incursion in all mandatory modes without the need for interrupted ventilation, special inspiratory flow, or occlusion patterns and maneuvers due to its software, which uses the least squares fitting (LSF) statistical method for measurement.^10^ Prior to extubation, the pulmonary oxygenation index was assessed using arterial gasometry. Within 48 hours of extubation, the patients were observed for their ventilatory conditions and the need for reintubation due to respiratory failure.

Once we had collected the necessary variables for analysis, we described the series, comparing sociodemographic, clinical, surgical, and pulmonary variables in two groups: patients who failed and those who succeeded in extubation. We evaluated a cut-off point for static lung compliance that best discriminates between patients who failed extubation and those who did not. Finally, we compared the variables between patients according to the cut-off point obtained for lung compliance.

### Statistical analysis

In order to describe the profile of the sample according to the variables under study, frequency tables were drawn up for the categorical variables, with absolute frequency (n) and percentage (%) values, and descriptive statistics for the continuous variables, with median and interquartile range. Pearson's chi-square test was used to compare the categorical variables between the two groups (with and without failure) or Fisher's exact test in the presence of expected values of less than 5%. The Mann-Whitney test was used to compare the numerical variables between the 2 groups due to the lack of normal distribution of most of the variables. The Kolmogorov-Smirnov test was used to verify the normality of the data. ROC curve analysis was used to assess a cut-off point for lung compliance that best discriminates between patients with and without extubation failure. To study the factors related to extubation failure, univariate and multiple Cox regression analysis was used, with a stepwise variable selection criterion. The level of significance adopted for the statistical tests was 5%, i.e., p<0.05.

## Results

### Overview of the study population

A total of 102 individuals were included in the sample. Their demographic, clinical, and surgical characteristics are shown in Table 1. The majority of the population was male, with a mean age of 59, non-smokers, and overweight. The most commonly performed procedures were myocardial revascularization, followed by valve replacement. The need for blood products (fresh frozen plasma and concentrated red blood cells) occurred in the majority of cases. The probability of death in the patient's first hour in the ICU, as measured by the SAPS score, was medium, as was the patient's severity index in the first 24 hours of hospitalization, as measured by the APACHE III score, which indicated a probability of death of 15%. In the SOFA score carried out at the time of the patient's admission to the ICU, all had some degree of organ dysfunction. In relation to the variables ventilation, the pulmonary oxygenation index verified by calculating Pao2/Fio2 was considered moderate according to the classification of the 2013 Brazilian Mechanical Ventilation Recommendations.^7^ The mean static lung static lung compliance was 42.55 cmH2O on admission to the ICU.

**Table 1:** Demographic, clínical, surgical, and ventilatory of the study population (n=102)

Variable	Results
Male% (n)	56.86( 58)
Female % ( n)	43.14 (44)
Age (Years)	61.50 (52.00-69.00)
Average body mass index ( Kg/m²)	25.30 (22.60-28.40)
Smokers % ( n)	47.06 (48)
Non-smokers % (n)	52.94 (54)
**Surgical data**	
Coronary artery bypass grafting % ( n)	48.04 (49)
Aortic valve replacement % ( n)	16.67 (17)
Mitral valve replacement % ( n)	13.73 (14)
Heart transplant % ( n)	6.86 ( 7)
Aortic prosthesis % ( n)	8.82 (9)
Closure of intra-valvar communication	2.94 (3)
Other surgeries % ( n)	3.00 (3)
Cardiopulmonary bypass time ( min)	88.50 (104.00-75.00) *
Nees for blood products % (n)	52,94% (54)
**Scores**	
APACHE	13.00 ( 16.00-10.00)
SAPS*	38.00 (32.00-43.00)
SOFA* de admission	5.00(4.00-7.00)
**Ventilatory data**	
Ventilation perfusion ratio ( Pao2/ Fio2)	169.00 (145.00-243.00)
Static lung compliance ( cmH2O)	4000 (35.00-49.00)

* APACHE II: Acute Physiology and Chronic Health Disease Classification System; SOFA: Sequential Organ Failure System; SAPS: Simplified Acute Physiology Score. Kolmogorov-Smirnov statistical test was used to verify the normality of the data; reference value p>0.05. Description: median and interquartile range

We observe in Figure 2 the proportion between groups of extubation success and extubation failure.

**Figure 2:** Graph referring to the outcome of extubations between the two groups: extubation success and failure within 48 hours.

Source: Author

### Comparative analysis between the groups: extubation failure versus extubation success

Table 2 provides a comparison of the demographic, clinical, and surgical variables between the two groups studied. The results show that there was a significant difference between those with and without extubation failure for females, who had more extubation failure than males, the scores, reintubated patients with a medium to high probability of death through the application of the APACHE and SAPS scores and with high organ dysfunction predicted through the SOFA score.

**Table 2:** Categorical and numerical comparison of demographic, surgical, and clinical characteristics between successful and unsuccessful extubation

Variables		Extubation failure (n=25)	Successful extubation (n=77)	p-Value
		% (n)	% (n)	
Male		32.00 (8)	35.06 (27)	
Female		68.00 (17)	64.94 (50)	
Chi-square test: X2=8.35; GL=1; p=0.004				
Smokers		60.00 (15)	42.86 (33)	
Non-smokers		40.00 (10)	57.14 (44)	
Chi-square test: X2=2.23; GL=1; p=0.136				
Need for blood products		64.00 (16)	49.35 (38)	
Chi-square test: X2=2.23; GL=1; p=0.136				
Surgery performed				
Myocardial revascularization		36.00 (9)	51.95 (40)	
Aortic valve replacement		20.00 (5)	15.58 (12)	
Mitral valve replacement		12.00 (3)	14.29 (11)	
Heart transplant		8.00 (2)	6.49 (5)	
Aortic prosthesis		16.00 (4)	6.49 (5)	
Fisher's exact test: p=0.463				
Age (years)		62.00 (53.00-69.00)	61.00 ( (52.00-68.00)	0.864
Body mass index (Kg/m²)		25.50 (23.80-26.40)	25.00 (22.40-28.70)	0.741
	APACHE II	15.00 (12.00-18.00)	12.00 (9.00-14.00)	0.004
	SAPS III	41.00 (38.00-47.00)	37.00 (31.00-42.00)	0.027
SOFA admission		6.00 (5.00-8.00)	5.00 (3.00-6.00)	0.011
CPB time (min)		91.00 (70.00-104.0)	88.00 (78.00-103.0)	0.861

P-value referring to the Mann-Whitney test for comparing values between the 2 groups. *APACHE II: Acute Physiology and Chronic Health Disease Classification System; SOFA: Sequential Organ Failure System; SAPS: Simplified Acute Physiology Score.

**Table 3:** Pulmonary variables between extubation failure and success

Variables	Extubation failure	Successful extubation	p-Value*
Static lung compliance	35.00 (30.00-40.00)	43.00 ( 38.00-43.00)	<0.001
Pao2/Fio2 ratio	156.0 (245.0-196.0)	183.0 (148.0-247.0)	0.161

P-value referring to the Mann-Whitney test for comparing values between the 2 groups. PaO2/ Fio2 ratio: Partial pressure of oxygen by fraction of inspired oxygen.

ROC curve analysis for static lung compliance versus extubation failure

Table 4 provides the results of the ROC (Receiver Operating Characteristic curve) analysis (figure 3) to assess a cut-off point for lung compliance as a predictor of extubation failure. The results showed that lung compliance had a significant area under the curve, with the cut-off point of compliance <41.0 cmH2O being associated with a higher probability of extubation failure.

**Table 4:** Results of the ROC*curve analysis for lung compliance versus extubation failure

Static lung compliance <41ml/cmH2O			
Variables	Failure extubation	Successful extubation	Total
% (n)	22.55% (23)	33.33% (34)	55.88% (57)
Cut-off point (Row)	40.35	59.65	
Probability of failure (Columns)	92.00	44.16	
**Static lung compliance** *≥* **41ml/cmH2O**			
% (n)	1.96% (2)	42.16% (43)	44.12 (45)
Cut-off point (Row)	4.44	95.56	
Probability of failure (Columns)	8.00	55.84	

Sensitivity (95%CI): 92.00% (72.50; 98.60) Specificity (95%CI): 55.84% (44.10; 67.00) Positive predictive value (95%CI): 40.35% (27.84; 54.16) Negative predictive value (95%CI): 95.56% (83.64; 99.23) Accuracy (95%CI): 64.71% (54.55; 73.74). Chi-Square test: X2=17.52; GL=1; p<0.001. ROC= Receiver Operating Characteristic Curve.

**Figure 3:** Area under the curve: AUC=0.799 (EP: 0.047); P<0.001. 95%CI: (0.706; 0.892) Cut-off point: compliance < 41.0 cmH2O.

Source: Autor.

### Cox regression analysis for extubation failure

Tables 5 and 6 below show the results of the Cox regression analysis to study the factors related to extubation failure. Initially, univariate analysis was carried out, followed by multiple analysis with Stepwise selection criteria.

**Table 5:** Results of the univariate Cox regression analysis for extubation failure (n=102)

Variable	Categories	p-Value	H.R.*	95%CI H.R.*
Age	Continuous variable	0,731	1,006	0,974 – 1,039
Gender	Male (ref.)	---	1.00	---
	Female	0,016	2,80	1,21 – 6,49
BMI*	Continuous Variable	0,950	1,002	0,936 – 1,073
PaO2/FiO2 ratio	Continuous Variable	0,235	0,996	0,990 – 1,002
CPB time (min)	Continuous Variable	0,916	0,999	0,981 – 1,017
Static lung compliance	Continuous Variable	<0,001	0,953	0,929 – 0,977
Static lung compliance (ROC* curve cut-off point)	≥41 (ref.)	---	1,00	---
	<41	0,003	9,08	2,14 – 38,50

HR (Hazard Ratio): Risk ratio for failure; (n=77 without failure and n=25 with failure). 95% HR = 95% confidence interval for the hazard ratio. Ref: reference level. BMI: body mass index; PaO2/ Fio2 ratio: Partial pressure of oxygen by fraction of inspired oxygen; CPB: cardiopulmonary bypass; ROC: Receiver Operating Characteristic Curve.

**Table 6:** –Results of the multiple Cox regression analysis for extubation failure (n=102)

Variable	Categories	p-value	H.R.*	95% CI H.R.*
1. Static lung compliance (ROC* curve cut-off point)	≥41 (ref.)	---	1.00	---
	<41	0.003	9.08	2.14 – 38.50

HR (Hazard Ratio) = Risk ratio for failure; (n=77 without failure and n=25 with failure). 95% HR = 95% confidence interval for the hazard ratio. Stepwise variable selection criterion. Ref: reference level.

The results of the multiple analysis showed the influence of the following factor on extubation failure: lung compliance (divided by the ROC curve cut-off point) presents a 9.1 times greater risk of failure for those with compliance < 41 cmH2O.

## Discussion

Failed extubation is one of the most frequent complications in the intensive care unit. According to the study by Ruan et al., 10% of extubations fail, and there is a need to return to mechanical ventilation within the first 48 hours.^11^ Failed extubation substantially increases the likelihood of death, as well as extending the length of stay in the ICU. Scott et al. concluded in their study that patients who fail extubation are seven times more likely to die and are 31 times more likely to have a prolonged hospital stay when compared to patients who succeed in extubation.^12^ In our study, 77 patients (75.49%) succeeded in extubation, while 25 patients (24.51%) failed extubation. These success and failure rates are similar to those of other studies, such as Danaga et al., who evaluated the extubation failure rate in a sample of 73 patients, where 58 (80.0%) remained extubated, and 15 (20.0%) failed and needed to return to mechanical ventilation.^13^ Souza and Lugon evaluated 109 patients; 65 patients (59.6%) were successfully extubated, but 36 (33%) died (8 patients had already been extubated), and the reintubation rate in this study was 10.7%.^14^ Although we have more successful extubations than failed ones, the consequences of reintubation are serious, given the high morbidity and mortality rate.

The study sample was composed predominantly of men, a characteristic that follows the trend of other studies such as that of Dordetto et al., who evaluated the demographic characteristics of 100 patients undergoing cardiac surgery, 56.0% of whom were men and 44.0% women.^15^ This may be justified by the fact that cardiovascular diseases are more lethal in women. According to the World Health Organization (WHO) (2018), heart disease accounts for one-third of the causes of death in women worldwide ^16^ due to the fact that the symptoms indicative of cardiovascular diseases are more generic in women, making diagnosis more difficult to start treatment and preventing them from reaching cardiovascular surgery.^17^ Despite the majority of men performing the procedure, the study found that women failed extubation more often than men. This fact may be justified by the study by Pereira et al., who pointed out that women's breathing pattern is predominantly thoracic because they have more mobile upper ribs, thus allowing for greater expansion despite having a smaller thoracic capacity due to their shorter sternum and more oblique upper thoracic opening.^18^ Therefore, due to thoracotomy with or without pleural incision, the intensity of surgical manipulation, and the number of pleural drains, women's thoracic expansibility is more compromised. Ambrozin et al., who researched aspects of pulmonary function in post-cardiac surgery patients, found that most women had reduced static lung compliance,^19^ resulting in worse gas exchange, which makes them more susceptible to acute respiratory failure and failure to extubate.

The patients’ mean age of 58.7 years did not have an impact on the outcome of extubation. This result can be justified by the study by Rocha et al., who concluded that patients over the age of 70 are more likely to develop respiratory complications in the postoperative period of cardiac surgery due to the physiological changes that occur in the aging process, changes in connective tissue increase the rigidity of the rib cage and reduce the elastic component of the lungs, influencing respiratory mechanics in addition to the decrease in respiratory muscle strength, added to this the thoracic impairment caused by the procedure, leads this population to be more susceptible to complications.^20,21^

The most common type of surgery performed was myocardial revascularization (48.04%), followed by valve replacement (30.43%). According to Data SUS (2018), around 23,000 cardiac surgeries were performed in Brazil, including valve repair and replacement and myocardial revascularization.^22^ We found no influence of the type of surgery on subsequent extubation failure. In the study by Assis et al., which assessed the influence of the type of surgery on postoperative complications in 57 patients, there were only two extubation failures, one in a patient undergoing myocardial revascularization and the other in a myocardial revascularization associated with valve replacement,^4^ which shows that the type of surgery is not a predominant factor in extubation failure.

Obesity makes the presence of atelectasis in the basal regions of the lung more common, reducing its compliance, and the presence of adipose tissue in the thoracic region increases air resistance.^23^ This condition has a negative impact on the outcome of extubation. Parlow et al. observed that their patients who met the criteria for obesity (body mass index (BMI) over 30) failed extubation 42.4% more often than overweight patients.^24^ This justifies the result found in our study, where the average patient was overweight (BMI 25.91), which did not significantly affect the outcome of extubation.

Although not statistically significant, smoking was related to a higher number of reintubations in this study. Ngaage D et al. justify this result by claiming that smokers develop pulmonary complications up to twice as often as non-smokers or former smokers, and as a result, these patients can remain on mechanical ventilation for up to six hours longer in the postoperative period of cardiac surgery.^25^ It is possible that early extubation in this population led to extubation failure.

The scores commonly used in intensive care units to predict patient severity and risk of death are APACHE II, SAPS, and the admission SOFA. We observed that the worst outcomes predicted by the scores were linked to patients who failed extubation. This result corroborates that of Shoji et al., who evaluated the rate of reintubation in patients undergoing cardiac surgery and found that 7.3% of the 119 patients evaluated in their study were reintubated during their stay in the ICU, of which 40.3% died.^26^

The PaO2/FiO2 ratio is used to determine the pulmonary oxygenation capacity of the patient to be extubated, and it is a criterion that this ratio be >200.^19^ Oxygenation is compromised in 20 to 90% of patients undergoing cardiac surgery with CPB.^15^ In our study, the PaO2/Fio2 ratio did not significantly influence the outcome of extubation, but it was lower in patients who failed extubation (178.4 ± 64.14) when compared to those who succeeded (203.0 ± 83.00).

Static lung compliance is an important factor in assessing lung mechanics, as its reduction increases respiratory work and can lead to respiratory failure. Compromised lung compliance in cardiac surgery is a consensus among studies on the subject and is attributed to inflammatory and mechanical factors in the pre-, intra-, and postoperative periods.^27^According to the Third Consensus on Mechanical Ventilation, compliance values considered normal are 60 to 100 ml/cmH2O.^15^ In this study, we observed that among the factors influencing extubation failure was critically reduced static lung compliance at the time of admission. It was possible to identify as a cut-off point the value of compliance < 41 ml/cmH2O, as the value associated with a 9.1 times greater likelihood of extubation failure among patients. This can be explained by a study by Cordeiro et al., which evaluated the impact of lung compliance on gas exchange in patients after cardiac surgery and concluded that the lower the patient's static lung compliance, the worse the gas exchange, generating acute respiratory failure and increasing the likelihood of reintubation.^28^

In an attempt to predict the success or failure of extubation, numerous variables to be assessed prior to extubation have been proposed over the years. However, the disparity between studies hinders an efficient assessment. In order to find reliable assessment parameters to predict the success of extubation, Baptistella et al. carried out a systematic review that included 43 articles, of which only two addressed lung compliance as a parameter to be used in the decision to extubate and none of the studies had patients after heart surgery as the study population.^29^ As proven in this study, impaired static lung compliance is a factor present among patients who fail extubation, and it is of great importance to include it in the assessment of patients after heart surgery.

### Limitations of the study

This study had some limitations. The fact that the study was carried out in a single hospital, with surgeries performed by the same medical team and standardized postoperative care, may not reflect the reality of other hospitals with different protocols and procedures. Not all the patients who underwent surgery were able to take part in the study, even if they failed extubation afterward, due to the fact that they were extubated in the operating room and it was not possible to collect the compliance value on the mechanical ventilator, which limited the sample size.

The patients included in our sample were not using the Swan-Ganz catheter, which made it impossible to accurately assess the hemodynamic profile and identify cardiogenic shock, for example, as a factor indicating reintubation, but what we do have is that no patient showed clinical signs of pre-extubation hemodynamic instability in the cases studied.

## Conclusion

We concluded that reduced static pulmonary compliance is a risk factor for extubation failure in post-cardiac surgery patients, with the cut-off point associated with greater failure being compliance <41ml/cmH2O, thus demonstrating that this is an important variable to be assessed prior to extubation in order to avoid possible reintubation and all the risks that this entails.
